# An Experimental Study on the Influence of Waste Tyre Metal Fibre on Asphalt Mixture’s Performance Properties

**DOI:** 10.3390/ma19050849

**Published:** 2026-02-25

**Authors:** Arsalaan Khan Yousafzai, Muslich Hartadi Sutanto, Nasir Khan, Jacob Adedayo Adedeji, Mongezi Mkhize, Nura Shehu Aliyu Yaro

**Affiliations:** 1 Department of Civil Engineering, Faculty of Civil, Agricultural & Mining Engineering, University of Engineering & Technology Peshawar, Peshawar 25120, Pakistan; 2 Department of Civil and Environmental Engineering, Universiti Teknologi PETRONAS, Seri Iskandar 32610, Malaysia; 3 Sustainable Environment and Transportation Research Group (SET-RG), Department of Civil Engineering, Midlands, Durban University of Technology, Private Bag X01, Scottsville, Pietermaritzburg 3021, South Africa

**Keywords:** waste tyre metal fibre (WTMF), asphalt mixtures, fatigue life, mechanical strength, moisture susceptibility, tensile strength, stiffness modulus

## Abstract

The disposal of waste tyres presents a significant environmental challenge, necessitating sustainable, high-value recycling solutions. This study explores the incorporation of waste tyre metal fibre (WTMF) into hot mix asphalt (HMA) to enhance mechanical performance while reducing landfill burden. WTMF-modified mixes containing 0%, 0.375%, 0.75%, 1.125%, and 1.50% fibre were evaluated through Marshall and volumetric testing, indirect tensile strength (ITS) and tensile strength ratio (TSR) for moisture damage, stiffness modulus at varying temperatures, and fatigue life under cyclic loading. Microscopic analysis revealed WTMF’s irregular, rough surface with microcracks and pits, aiding crack-bridging and stress transfer. Marshall testing showed that the optimum binder content of WTMF-modified mixtures was approximately 5% higher than that of the control (conventional HMA without WTMF); however, stability decreased while flow increased, resulting in a reduced Marshall quotient due to fibre conglomeration affecting porosity and bulk specific gravity. ITS results indicated that the control mixture exhibited the highest cracking resistance, whereas WTMF-modified mixtures demonstrated improved moisture resistance (TSR > 80%). The maximum improvement was observed at 0.75% WTMF-induced HMA, with an 11% increase in TSR, while a slight reduction of 2.4% occurred at 1.50% WTMF-induced HMA. Stiffness testing showed that the mixture containing 0.375% WTMF achieved the highest modulus, exhibiting up to a 70% increase at 5 °C and more than a twofold increase at elevated temperatures compared to the control mixture. With increasing temperature, stiffness decreased by approximately 84% for the control mixture and 80% for the 0.375% WTMF-modified mixture. Fatigue analysis showed that the control mixture achieved a fatigue life of 115,529 loading cycles at low stress, followed by substantial reductions in fatigue life with increasing stress levels, whereas moderate WTMF contents improved strain performance; however, excessive fibre content increased permanent deformation under high stress. Stress- and strain-based empirical power-law relationships were established for predicting the fatigue life of each investigated mixture. Results demonstrate that WTMF’s controlled dosage within the optimum range of 0.375 to 0.75% has the potential to improve HMA’s performance indicators, offering a sustainable recycling pathway for waste tyres.

## 1. Introduction

Asphalt pavements are extensively used worldwide. Since its service life is influenced by a variety of external factors, several modifications are being made in an effort to make asphalt pavements more sustainable [1,2,3]. This modification is carried out by adding various reinforcement materials to traditional asphalt mixes [4]. The use of metallic fibres offers several advantages, like enhancing the electrical conductivity to enable self-sensing ability in traditional asphalt mixes that can be further used for traffic monitoring, pavement damage sensing, guidance of autonomous vehicles, non-destructive testing, snow melting and de-icing, and many other applications [5]. Several types of metallic modifiers have been studied by researchers to modify asphalt mixes. Some examples include carbonyl iron powder, iron tailing, steel, copper wire, steel slag, and aluminum metal fibre [6]. Steel fibres, including steel wool fibre (SWF), are among the most commonly used modifiers in asphalt mixtures, with previous studies reporting intrinsic tensile strengths of individual fibres of up to 502 MPa [7,8,9]. Wang et al. [8] reported significantly increased stability, rutting resistance, and tensile strength of SWF-modified asphalt specimens. This is because well-distributed steel fibres enable the mixture to transfer higher stresses through the formation of a complex three-dimensional reinforcing structure. Similar findings were reported by Shaffie et al. [10], who observed improved stability and flow properties with the incorporation of steel fibres in asphalt mixtures. Furthermore, the same study reported improvement in dynamic creep and moisture susceptibility because of adding steel fibre.

Researchers have mostly used virgin metallic modifiers in asphalt to enhance its sustainability and performance, but these materials are often costly. Consequently, the utilization of waste metallic materials as modifiers for asphalt modification can help reduce the cost involved, maintain the pavement’s mechanical performance needs, and address the issue of environmental sustainability. In such conditions, waste tyres could be a viable option. Since the global tyre production is expected to exceed 2.7 billion units by 2027 [11,12], these are considered to be one of the largest landfill wastes, which pose significant environmental challenges [13]. According to Abdulrhman et al. and Rogachuk et al. [14,15], around 1.5 billion tyres are wasted after their end-of-life annually. Moreover, according to Valentini et al. [16], a passenger car tyre and a commercial truck tyre are composed of 11 to 21% by weight of steel wire alone, respectively. Alongside others, this material is used to reinforce the tyre. Given this data, a safe estimate shows that more than 5 million tonnes of steel wire are discarded from these tyres annually. Hence, there is a huge amount of steel wire generated from waste tyres, and thus, there is a substantial opportunity for recycling and sustainable material recovery. Although research has been done on the use of ground tyre rubber and tyre fabric as well [17], few studies have shown the utilization of steel wires extracted from waste tyres. Therefore, it is both important and timely to utilize such waste materials to reduce the overall volume of waste disposed of in landfill sites.

It is well-known that mechanical performance is the key parameter to be assessed before the asphalt is implemented on site. It means that maintaining the needed levels of strength, moisture susceptibility and stiffness in new asphalt mixes is a challenge for researchers. Similarly, assessment of the asphalt mix’s resistance to fatigue failure is also important. Moreover, the wise utilization of waste tyres and the production of innovative asphalt materials to cater to sustainable development goals and higher traffic load requirements is a challenge. Although previous studies have investigated the use of conventional steel fibres and recycled metallic fibres in asphalt mixtures, limited attention has been given to waste tyre metal fibre (WTMF) recovered directly from end-of-life tyres. Unlike commercially manufactured fibres, WTMF exhibits irregular geometry and surface characteristics resulting from the tyre shredding and extraction process, which may influence fibre–binder interaction and reinforcement behaviour. Furthermore, existing studies on tyre-derived fibres often focus on conductive properties, with comparatively fewer investigations addressing their influence on key mechanical performance characteristics of asphalt mixtures. Therefore, this study aims to systematically evaluate the effect of WTMF on the performance properties of asphalt mixtures, thereby providing insight into its potential as a sustainable and practical reinforcement material. The primary objective of this study is to evaluate the influence of WTMF on the mechanical performance indicators of asphalt mixtures compared to a conventional control mixture. For this purpose, this study uses WTMF in proportions of 0% (control), 0.375% (Series A), 0.75% (Series B), 1.125% (Series C), and 1.5% (Series D) as modifiers, along with varying optimum contents of bitumen for each mix to investigate their effect on the tensile strength, moisture susceptibility, stiffness behaviour, and the fatigue life of these modified HMA mixtures. WTMF content is expressed as a percentage by mass of the total asphalt mixture. The study explores the potential application of WTMF as a sustainable and cost-effective reinforcement in flexible pavement design. This study also demonstrates the development of modified asphalt mixtures with enhanced performance through the use of an optimum WTMF dosage. Lastly, this study serves as an effort to reduce tyre landfill waste to contribute towards environmental sustainability.

**Figure 1:**
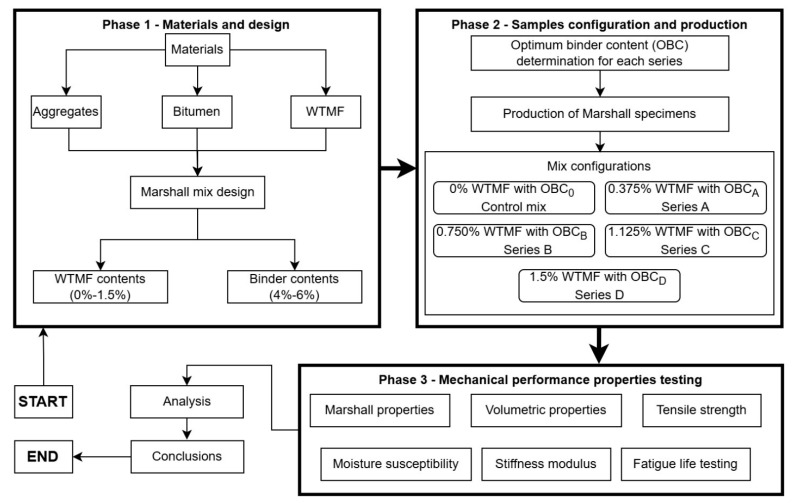
Phase-wise flow chart of the experimental program. Bold boxes and arrows represent transition from one phase to the next.

The selection of experimental parameters and WTMF content levels in this study was guided by a comprehensive review of previous research on fibre-modified asphalt mixtures, as summarized in Table 1. Reported fibre contents in the literature generally lie within a limited range, beyond which workability and fibre dispersion issues have been observed. Accordingly, WTMF dosages as described above were selected to enable a systematic evaluation of reinforcement effects while maintaining practical mixability. These dosage levels were further validated through preliminary trial mixing to ensure uniform fibre distribution. The overall scheme of this study is outlined in Figure 1. First, the raw materials are characterized for their basic properties. Next, the Marshall mix design is performed for the pre-determined contents of WTMF and binder for assessing the rheology and volumetric properties of each mix, after which the optimum content of the binder is obtained to be used for subsequent sample preparation. Then, the selected performance testing experiments are done for each mix series, and the results are analyzed afterwards. The subsequent sections provide details about the materials and experimental program, results and discussion, and finally, the conclusions are drawn. The novelty of this study lies in the systematic experimental evaluation of waste tyre metal fibre as a reinforcement material in asphalt mixtures, with emphasis on performance properties rather than conductivity, and in explicitly differentiating its behaviour from that of conventional steel fibres.

## 2. Materials and Methodology

### 2.1. Material Characterization

**Figure 2:**
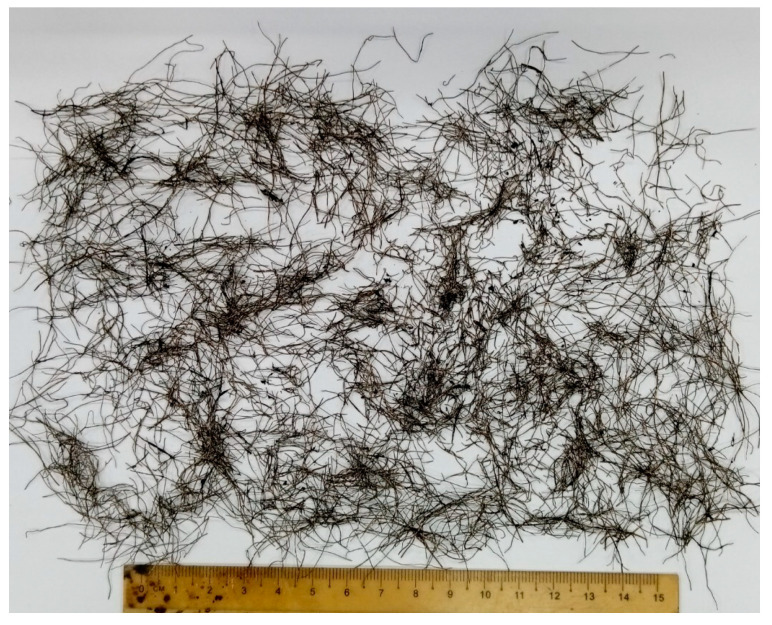
Waste tyre metal fibre used in this research.

**Figure 3:**
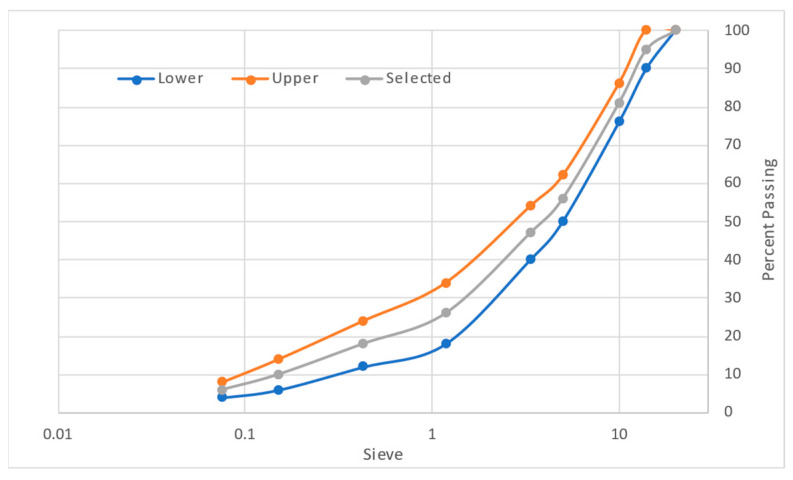
JKR’s aggregate gradation limits.

The research was carried out using local highway construction materials available in Perak, Malaysia. Aggregates were acquired from Sunway Quarry Industries Sdn. Bhd. (Subang Jaya, Malaysia), whereas 60/70 penetration grade bitumen was acquired from Viking Asphalt Sdn. Bhd. (Johor, Malaysia), keeping in view the climate in Malaysia. Table 2 shows the material characteristics of aggregates and bitumen used in the study. The naked eye appearance of WTMF, which is used as the primary asphalt modifier in the research, is shown in Figure 2. The content of WTMF is selected so as to conform to previous research studies, as given in Table 1, as well as trial laboratory testing to address the issue of agglomeration, heterogeneous mixing, and inadequate compaction at higher contents. Moreover, Malaysia’s Jabatan Kerja Raya (JKR) specifications were adopted for the Marshall mix design [28]. That is, an asphaltic mix with a maximum nominal aggregate size of 14 mm (i.e., AC-14) was adopted for aggregate gradation, representing the pavement’s wearing course. The aggregates were sieved accordingly through the specified sieve sizes to achieve the required combinations based on particle size. Figure 3 shows the aggregates’ gradation limits utilized in this research as per the specification used.

Regarding the characterization of WTMF, Field Emission Scanning Electron Microscopy (FESEM) was utilized using Carl-Zeiss Supra 55VP (Carl Zeiss AG, Oberkochen, Germany), which is a commonly employed technique to analyze the morphology of the modifiers used in asphalt mixes [4,29,30].

### 2.2. Preparation and Testing of Samples

**Figure 4:**
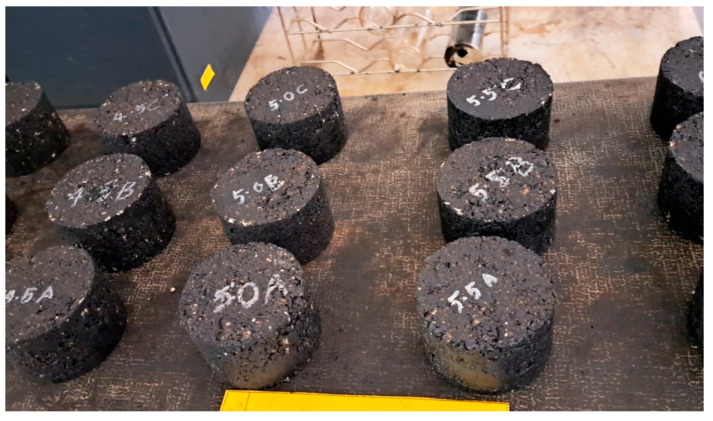
Marshall specimens prepared for OBC determination.

The initial stage was to determine the optimum binder content (OBC) for the control samples as well as each modified mixture type with the specific content of WTMF. The Marshall mix design procedure was adopted for determining the OBC, whose objective is to optimize the quantity of binder needed for a specific mix type to make a durable mix. The OBC was determined for the control as well as WTMF-modified mixes in accordance with the technique established by the Asphalt Institute [31]. A range of 4 to 6% bitumen was selected with an increment of 0.5%. The parameters taken into consideration for determining the OBC were Marshall stability, flow, voids in mineral aggregate (VMA), porosity, bulk specific gravity (G_mb_), and voids filled with binder (VFB). In the first phase, various proportions of bitumen (i.e., 4%, 4.5%, 5%, 5.5%, and 6%) were used to calculate the OBC for each mix series (control and Series A–D). The acquired OBC value for each type of mix was different from the other because of varying contents of WTMF in each mix. Marshall specimens were produced in accordance with ASTM D6926-20 [32]. The aggregate composition of coarse and fine aggregates and mineral filler was 44%, 50%, and 6%, respectively, to achieve the target gradation. These graded aggregates were first placed in an oven at ~150 °C for at least 15 min to ensure that any moisture contained in them is completely removed. Pre-weighed dry aggregates were placed in a container, followed by bitumen at the optimum binder content, and WTMF was added gradually during mixing to ensure uniform distribution. Bitumen was pre-heated at 180 ± 5 °C, and the combined mixture was mixed at 105 ± 15 °C for optimal workability prior to compaction. The dry mixing process was adopted for blending the fibres in the mix of dry aggregates and binder, during which WTMF were added in intervals of small quantities to ensure homogeneous mixing. A total of 75 samples were prepared for the determination of Marshall parameters and OBC for the control and modified mixes. Figure 4 shows the samples prepared and placed overnight to allow them to cool down to room temperature.

**Figure 5:**
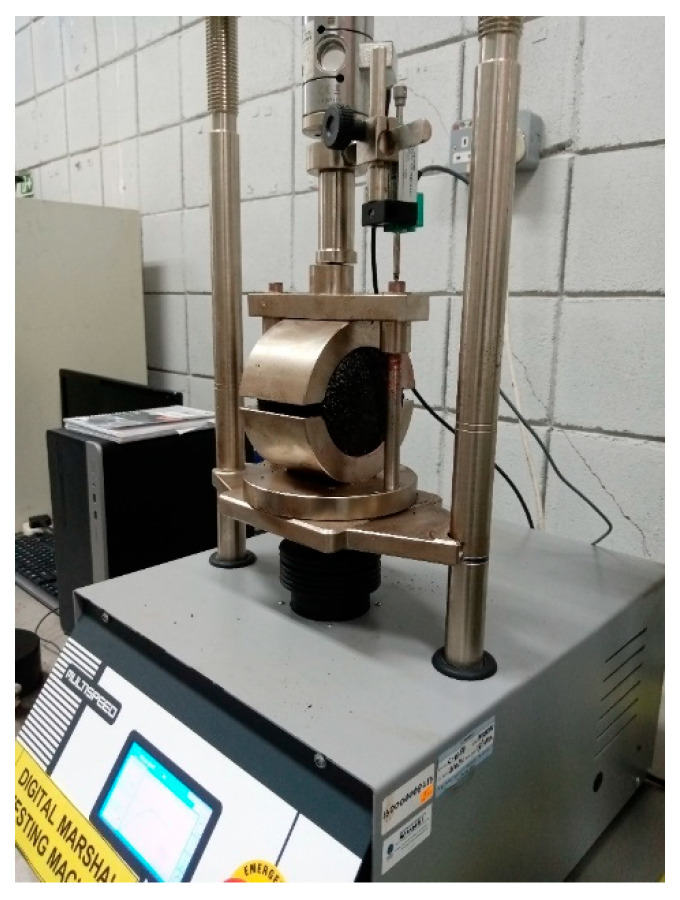
Marshall testing apparatus used in the study.

Once the samples were prepared, the assessment for bituminous mixture’s resistance to deformation and exposure to traffic load was done using Marshall testing, which was carried out in accordance with ASTM D6927-15 [33] standard using the equipment as shown in Figure 5.

### 2.3. Tensile Strength and Moisture Susceptibility

**Figure 6:**
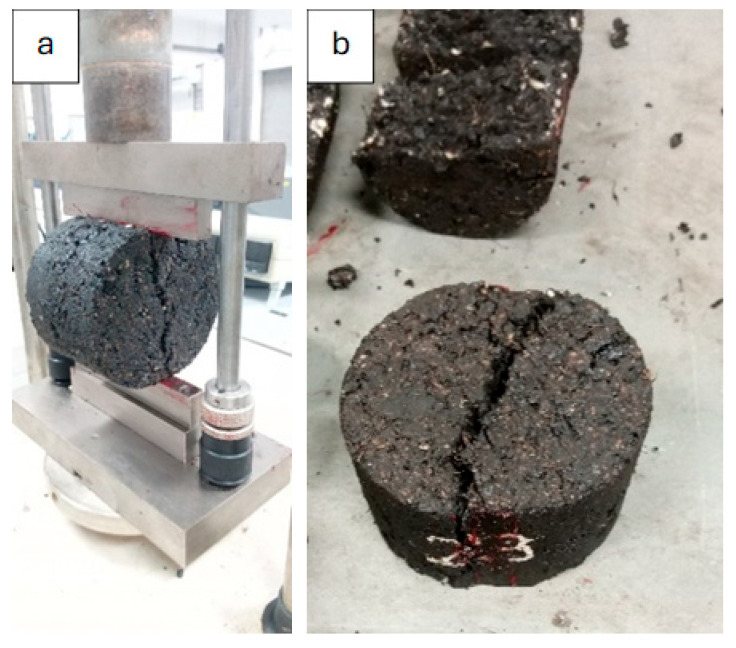
(**a**) Load application mechanism via UTM. (**b**) Sample after ITS test.

The tensile strength testing is a widely used parameter for assessing the asphalt’s performance properties and cracking resistance, primarily due to its simplicity and cost-effectiveness [34]. In addition, moisture damage/stripping is one of the main causes of distress in asphalt pavement as it shortens its design life and increases the maintenance cost [35,36]. It also reduces the ability of bitumen to bond with the aggregate surface due to the infiltration of moisture [37,38]. It is usually caused by rainwater that penetrates the mixture through cracks. It can also happen due to the incompatibility of the asphalt binder with aggregates. If once initiated, it can accelerate pavement distress such as cracking and rutting due to weakened adhesion between the two elements [39,40,41]. In this study, the effect of WTMF on the tensile strength and moisture damage resistance of modified asphalt mixtures was investigated. The indirect tensile strength (ITS) and moisture susceptibility tests of asphalt mixtures were done on Marshall specimens as per ASTM D6931-17 [42] and ASTM D4867-14 [43] standards, respectively. Six samples for each mix were prepared, half of which were tested under dry conditions, while the remaining ones were tested under wet conditions to simulate the effect of moisture damage. Monotonic uniaxial compressive ramp load was applied via a universal testing machine (UTM) with a maximum force capacity of 30 kN, as shown in Figure 6a, at a constant displacement rate of 51 mm/min (2 in./min) until fracture. Transverse tensile load in the sample’s horizontal direction is indirectly produced because of the vertically applied compressive load using this mechanism. The tensile strength of the specimen was then calculated using Equation (1) [44].

(1) $$ITS=\frac{2000\times P}{\pi \times t\times D}$$

Here, ITS is the indirect tensile strength (kPa), P is the maximum applied force (N), t is the sample height (mm) measured just before conducting the test, and D is the sample diameter (mm). Moreover, the moisture resistance of the asphalt sample is represented by its tensile strength ratio (TSR), usually measured in percentage [30]. It is the ratio of the ITS values for both the dry and moisture-induced samples, as given in Equation (2). The minimum value of TSR should be 80%, with a higher value indicating greater resistance to moisture damage [45]. Conversely, a low TSR would suggest the mixture is more susceptible to moisture damage, which could lead to reduced pavement performance and premature failures, such as rutting, cracking, or potholing. For preparing the conditioned samples, the Marshall specimens were moisture-conditioned by soaking them in distilled water at 60 ± 1 °C for 24 h. Then, the temperature of the moisture-conditioned samples was readjusted by soaking them in a water bath for one hour at 25 ± 1 °C. Figure 6b shows the samples after the test was performed, in which significant cracking can be observed. It can be observed that the samples have not been split completely in the test because of the fibres in the mastic that kept the two halves intact.

(2) $$TSR=\left(\frac{{ITS}_{controlled}}{{ITS}_{conditioned}}\right)\times 100$$

### 2.4. Stiffness ModulusTesting

The stiffness modulus of asphalt concrete mixtures was determined by the indirect tensile stiffness modulus (ITSM) test using BS EN 12697-26:2004 [46] and ASTM D7369-20 [47]. Besides ITS and moisture susceptibility, the stiffness modulus is a fundamental material parameter for the assessment of asphalt’s viscoelastic performance under traffic loading and is considered to be an input for the mechanistic–empirical design of pavements [48]. It is calculated using Equation (3), where S_m_ is the stiffness modulus (MPa), P is the applied cyclic peak load (N), ν is the Poisson’s ratio (assumed to be 0.35), H is the recoverable horizontal deformation (mm), and h is the specimen thickness (mm).

(3) $$S_{m}=P\times \frac{\nu +0.27}{H\times h}$$

**Figure 7:**
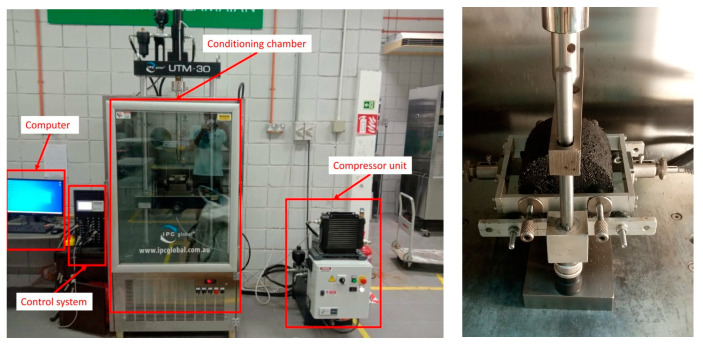
(**left**) UTM used for ITSM testing. (**right**) Sample’s placement assembly.

A series of ITSM tests was carried out to determine the stiffness of the modified asphalt mixes with predetermined WTMF contents at four different temperatures, i.e., 5, 15, 20, and 25 °C. Three Marshall samples from each mixture type were tested at each temperature, and the average results at each temperature were used for the analysis. The IPC Global UTM-30 Servo-Hydraulic Universal Testing Machine, Model 79-PV70B12/I2 from Control Group, Liscate, Italy, was used in the testing, as shown in Figure 7(left). Haversine waveform cyclic compression load pulses were applied during the test to achieve the target horizontal deformation. Moreover, the ITSM testing was carried out on two perpendicular diametrical axes of each sample to achieve test results in the 0° and 90° directions, and the average was used in this study. Figure 7(right) shows the sample’s placement assembly inside the equipment.

### 2.5. Fatigue Testing

**Figure 8:**
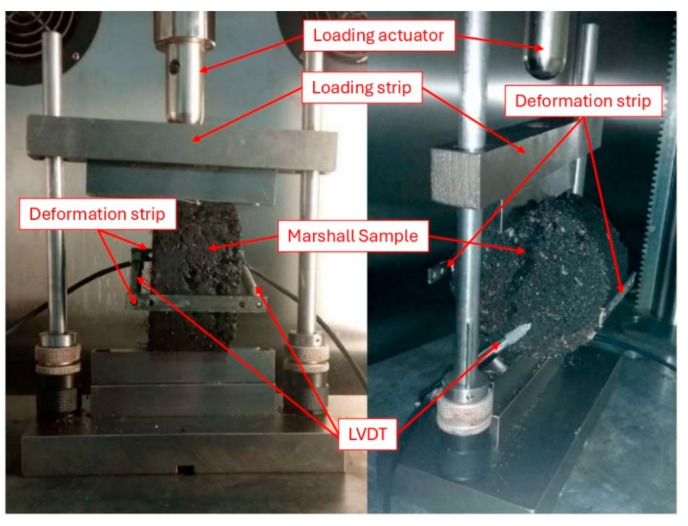
Front (**left**) and side (**right**) views of the ITFT setup for a Marshall sample of the study.

Fatigue is the reduction in the strength of a material under repeated loading. Asphalt fatigue cracking is a significant problem in pavements that has remained the centre of attention and been studied by various researchers [49,50,51]. Fatigue damage arises in the wearing course, which is exposed directly to the traffic loads. Moreover, the performance of asphalt mixtures can be more accurately predicted when their resistance to fatigue is known. Hence, the indirect tensile fatigue testing (ITFT) was used to estimate the fatigue life of the asphalt mixes under consideration under repeated traffic loading. Marshall specimens with a 900 g weight of aggregate batches were prepared and tested for fatigue test BS EN 12697-24 [52]. In order to get the best fatigue fit models, three stress levels were chosen as mentioned in the same test standard. The stress levels used in the indirect tensile fatigue test (ITFT) were determined based on ratios of the indirect tensile strength (ITS) values of the specimens [53,54]. Accordingly, in the current study, stress levels of 25% (low), 33% (medium), and 40% (high) of respective ITS values of the control mixtures were selected for conducting ITFT, which came out to be 250 kPa, 350 kPa, and 450 kPa, respectively. This approach is consistent with the methodologies employed by Mullapudi et al. [55] and Cui et al. [56]. A minimum of three replicate specimens from each mix type were tested at each cyclic tensile stress level. Moreover, as recommended by the test standard, a stress level of 250 kPa was selected because this value was found to be suitable for most of the mixtures. The test is supposed to continue until either the complete fracture of the specimens or deformation reaches 9 mm, as per BS EN 12697-24 [52]; however, in this study, failure consistently occurred through fracture before the deformation limit was reached. The maximum tensile stress and the horizontal tensile strain as a function of stress and stiffness of the mixture can be obtained from Equations (4) and (5). In these equations, $\sigma_{x(max)}$ is the maximum tensile stress at the specimen centre (MPa), $P_{max}$ is the peak applied load (N), d is the specimen diameter (mm), t is the specimen thickness (mm), $\varepsilon_{x(max)}$ is the maximum tensile strain at the specimen centre, $S_{m}$ is the specimen’s stiffness modulus (MPa), and μ is Poisson’s ratio, usually assumed to be 0.35 for 20 °C testing temperature. Three specimens from each mixture and at each stress level were tested for ITFT, and the average of the results was used for analysis. The test setup for ITFT is shown in Figure 8.

(4) $$\sigma_{x(max)}=\frac{2\times P_{max}}{\pi \times d\times t}$$

(5) $$\varepsilon_{x(max)}=\frac{\sigma_{x(max)}}{S_{m}}\times (1+3\mu )$$

It is important to note that total horizontal micro-strain (THµε) is composed of both resilient (Rµε) and permanent micro-strain (Pµε). Among these, resilient micro-strain is the most relevant parameter for fatigue analysis. This is because it represents the recoverable strain after each load cycle, reflecting the elastic behaviour of the asphalt mixture. Secondly, it is commonly used in strain-based fatigue models for predicting fatigue life. Thirdly, it facilitates the comparison of asphalt mixtures in terms of flexibility and resistance to fatigue cracking. In contrast, deformation analysis typically focuses on permanent deformation, which reflects accumulated, non-recoverable strain leading to material failure over time. Permanent deformation is crucial in assessing rutting resistance and long-term structural integrity of asphalt pavements. Results obtained regarding these parameters are discussed in detail in the next subsections.

## 3. Results and Discussion

### 3.1. WTMF Characterization

The microstructural and compositional analysis of WTMF was conducted using Scanning Electron Microscopy (SEM) coupled with Energy-Dispersive Spectroscopy (EDS). EDS is a technique used to determine the elemental composition of a sample by detecting X-rays emitted from the material when it is bombarded with an electron beam inside an SEM. These SEM images provide insights into the fibre’s morphology, surface characteristics, and dimensional consistency, while the EDS spectrum identifies its elemental composition.

**Figure 9:**
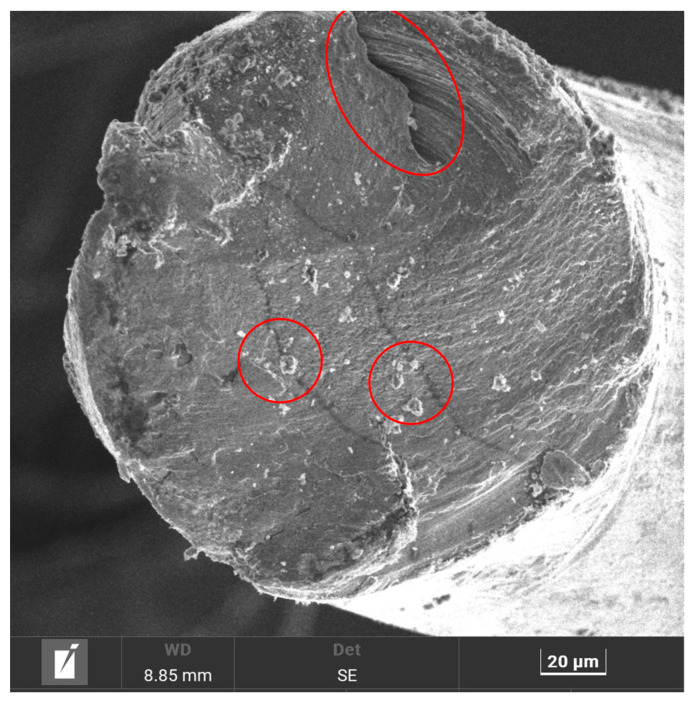
SEM image @1000× of the diametrical face of WTMF. Red encircled areas highlight the observed surface irregularities.

Figure 9 presents the SEM image of the diametrical face of WTMF at 1000× magnification, revealing a rough and irregular surface morphology. SEM analysis was performed on WTMF samples prior to mechanical testing to characterize fibre geometry and surface texture. The observed surface irregularities are primarily attributed to the mechanical shredding and extraction processes used during fibre recovery from waste tyres. The fibre’s cross-sectional surface exhibits irregularities, which indicate possible mechanical wear, material degradation, or prior exposure to environmental conditions. These surface imperfections may play a significant role in influencing the fibre’s bonding behaviour within the asphalt mastic. The presence of these irregularities suggests that the fibre may have undergone prior stress or thermal effects during the tyre shredding and fibre extraction process, which could affect its mechanical performance when embedded in concrete. On the other hand, these surface irregularities may enhance the mechanical interlocking between the fibre and the surrounding mastic, potentially improving its pull-out resistance. However, excessive roughness or deep pits could also lead to stress concentration points, impacting the fibre’s performance under load application. These characteristics may influence the fibre’s mechanical interlocking with the surrounding asphalt mastic, affecting its overall performance.

**Figure 10:**
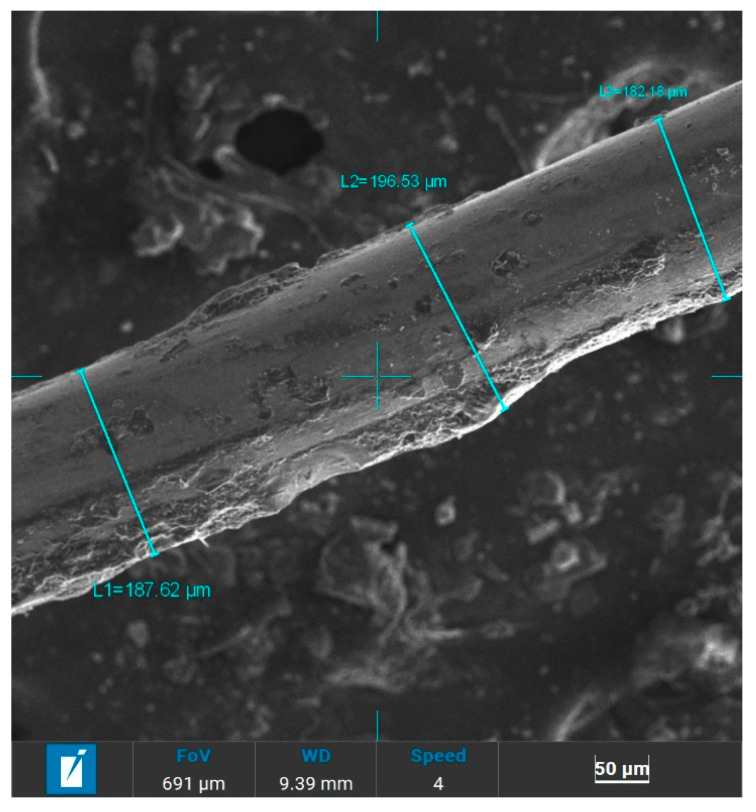
SEM image @300× of the longitudinal face of WTMF.

Figure 10 illustrates the longitudinal face of WTMF at 300× magnification, where three diameter measurements were taken to assess its dimensional consistency. The recorded diameters were 187.62 μm, 196.53 μm, and 182.18 μm, resulting in an average diameter of 188.78 μm. The slight variation in fibre diameter indicates non-uniformity, which may result from manufacturing inconsistencies or surface wear. Such non-uniformity can hinder uniform dispersion in the asphalt mastic, as fibres with larger diameters may clump or entangle, while thinner fibres may distribute more easily. This, in turn, can influence the efficiency of load transfer and reinforcement within the asphalt mixture under applied stress.

The aspect ratio is crucial in determining the reinforcing effectiveness of the fibre. It was based on the fibre length, which was observed to vary between 3 mm and 9 mm randomly, so an average length of 6 mm (6000 μm) was considered for calculating the aspect ratio. The length of the fibre was determined after measuring the length of many random pieces of the fibre and then averaging them. With an average diameter of 188.78 μm, the aspect ratio was determined to be approximately 32. This high aspect ratio indicates that the fibres are long and thin, desirable for asphalt mix in reinforcing its structure and improving the interfacial bonding. Such a high value of the aspect ratio generally enhances the mechanical interlocking and crack-bridging ability of the fibre in asphalt, contributing to improved performance.

**Figure 11:**
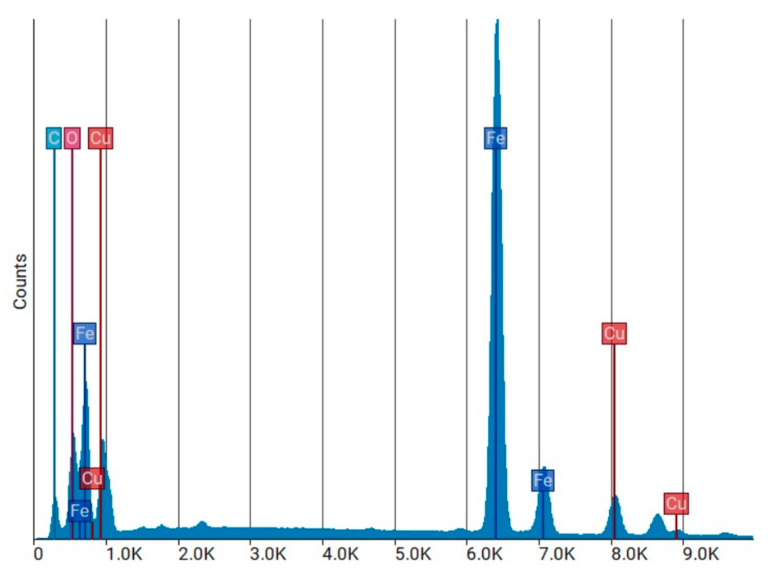
EDS elemental spectrum.

The EDS elemental spectrum (Figure 11) shows the elements detected in the WTMF sample. The *X*-axis represents the energy of emitted X-rays (in keV), and the *Y*-axis indicates the intensity (counts) of detected X-rays. Each element produces characteristic peaks at specific energies, allowing for identification, while the peak height reflects its relative abundance in the scanned area. Quantitative analysis of the elements, including atomic and weight fractions, is summarized in Table 3.

The results indicate that iron (Fe) is the dominant element, with a weight percentage of 51.65%, confirming the metallic nature of the fibre. The presence of oxygen (12.13% Wt.%) suggests surface oxidation, likely due to environmental exposure or corrosion. Carbon (C) is present in a significant amount (57.32% At.%), which may be attributed to residual rubber, coatings, or polymeric components of the original tyre reinforcement. It should be noted that EDS analysis provides elemental composition only and cannot identify molecular or polymeric phases. The carbon detected in WTMF is mainly associated with the carbon steel composition of the fibres and minor surface carbonaceous residues from tyre processing, rather than the presence of rubber or polymeric material. The SEM and EDS findings confirm that WTMF primarily consists of iron, with additional contributions from carbon and oxygen. The presence of an oxide layer could influence the bonding efficiency of the fibre within cementitious materials, while the high carbon content suggests remnants of the original rubber or polymer coatings. These factors are crucial in understanding the performance of WTMF as a reinforcement material in asphalt modification.

### 3.2. Marshall and Volumetric Properties

The Marshall parameters are one of the most widely used and important aspects of pavement performance, since they provide critical insights into the performance of asphalt mixes. Table 4 illustrates the results for Marshall parameters and volumetric properties for the control and WTMF-modified mixtures.

**Figure 12:**
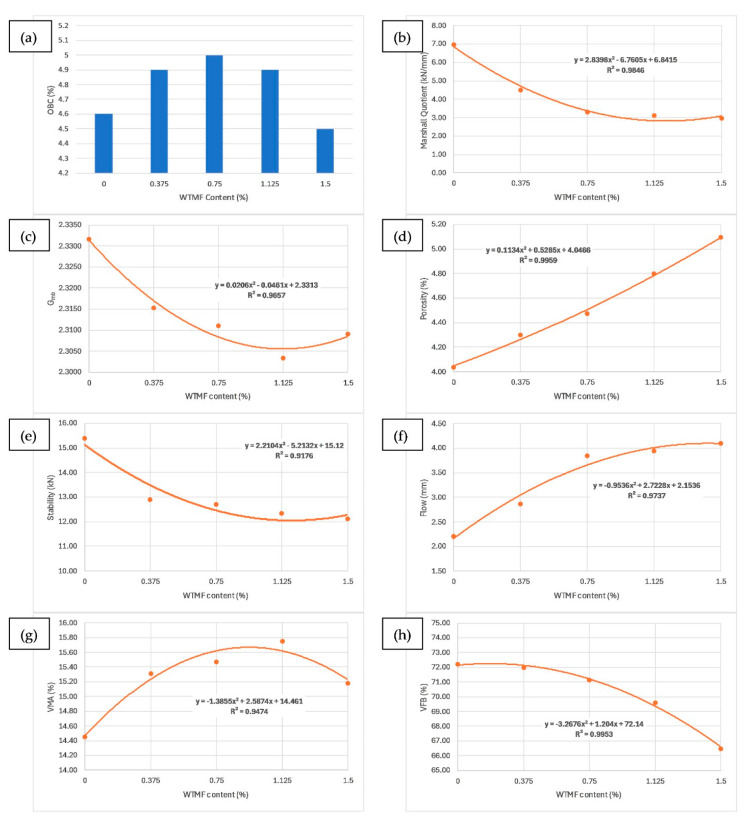
Effect of WTMF on (**a**) OBC, (**b**) Marshall quotient, (**c**) bulk specific gravity, (**d**) porosity, (**e**) Marshall stability, (**f**) flow, (**g**) VMA, (**h**) VFB.

From Table 3 above and Figure 12a, it can be observed that the OBC varies for each mix type due to varying proportions of WTMF. The highest OBC was found for the Series B mix, with a WTMF content of 0.750%. Moreover, a decline in the trend of the Marshall quotient (MQ) was also observed with the addition of WTMF in the mixes, as shown in Figure 12b. Figure 12c represents the effect of fibre content on the bulk specific gravity of all the mix types, which shows a declining effect with an increase in WTMF content. On the other hand, porosity was observed to increase linearly with an increase in WTMF content, as presented in Figure 12d. This increase is due to the conglomeration of fibre during the mixing and compaction process. Moreover, the overall surface area of the asphalt mastic increases with the incorporation of fibre, due to which it is logical to see an increase in the porosity of the mixes with increasing fibre content. This leads to a less dense mix, affecting the Marshall stability of the mix. Figure 12e shows the effect of WTMF content on Marshall stability, showing a decline in stability with an increase in fibre content, further supporting the relationship observed between air voids and fibre content. Similarly, Figure 12f shows the effect of WTMF content on the flow of asphalt mixes, showing an increasing trend in flow with an increase in fibre content. Initially, the slope is steeper, and then it stabilizes with an increase in fibre content. Similar results were reported by Al-Bdairi et al. [57]. The figure represents an inverse relationship between Marshall stability and flow, with values ranging from 6.96 kN/mm for control samples to 2.95 kN/mm for the maximum WTMF content. Since it is a parameter used to indicate the resistance of the asphalt mix to deformation, it indicates that the WTMF-modified mixtures are less rigid compared to the control mixture, which might be due to a lower amount of internal friction in mixtures containing WTMF.

The maximum value of VMA was observed for content between Series B and Series C, as shown in Figure 12g above. It can also be observed that there is a direct impact of VMA on the OBC, since more bitumen can be accommodated in the mix with more VMA. However, for Series D, a decline in OBC also resulted in lower VMA. Similarly, as depicted in Figure 12h, the VFB decreased from 72% to 66.5% with the addition of fibre, due to absorption of bitumen by the increased surface area of the fibre content. The same argument justifies the reduction in OBC for the Series D mixture.

### 3.3. ITS and TSR

**Figure 13:**
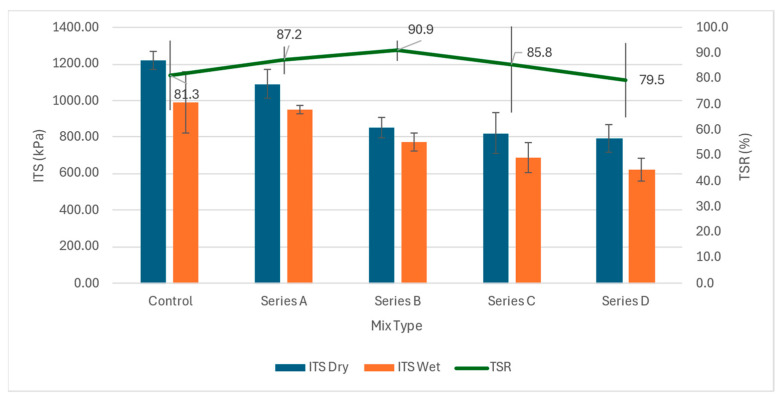
ITS and TSR test results.

ITS testing was performed for both the dry samples and the wet conditioned samples to assess the performance of the modified asphalt exposed to moisture, and to assess the level of moisture resistance. Figure 13 illustrates the ITS and TSR values for both categories of HMA blends produced with varying ratios of WTM fibre prepared at optimum contents of bitumen. As seen from the results, the pattern of ITS results for the wet samples is like that of the dry ones. Moreover, the use of WTMF increases the TSR, thereby reducing the moisture susceptibility of the asphalt mixture.

The results indicated that HMA blends containing WTM fibre in the samples had somewhat lower cracking resistance when compared to the control blends. Similar results were reported by Alvaro et al. [58], where the authors used metallic fibres from recycled tyres in asphalt mixtures to assess crack-healing potential. Similarly, another study found that ITS peaked at a low fibre content and declined at higher dosages [59]. Similar results were also reported by Yang et al. [60]. Regarding the TSR values, it was found to be maximum for the Series B mix. This is related to the shape and surface area of the metal fibre being incorporated; this property improves the tensile strength and resistance, and reinforces the overall structure of the mastic. Afterwards, TSR for Series C and Series D were reduced, in which the value for Series D samples was even less than that of control samples because of excessive use of WTMF in the sample mix. This finding is similar to the study by Shaffie et al. [10].

### 3.4. Stiffness Modulus

**Figure 14:**
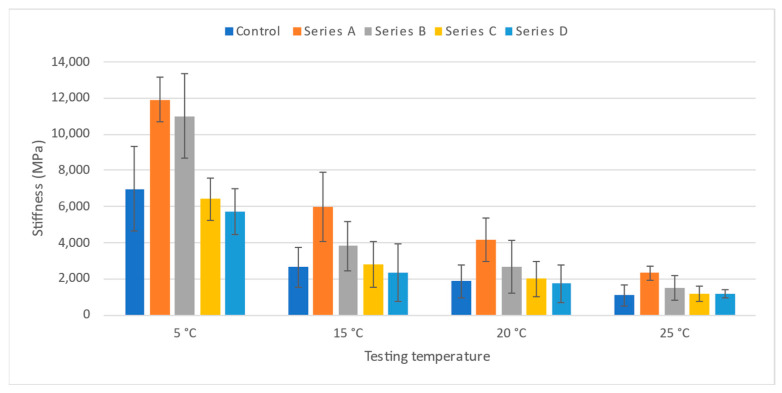
ITSM test results.

The stiffness modulus of asphalt is an important input parameter for the structural design of pavement layers, since it indicates how the traffic load is distributed effectively to the underlying layers. This test is also used in ranking different asphalt mixtures based on stiffness. The stiffness modulus is also used to evaluate the structural behaviour of pavement. The temperature-wise average stiffness modulus of all mixtures is presented in Figure 14 to better understand the stiffness behaviour of each type of modified asphalt compared with conventional asphalt mixtures. The stiffness values for all mixes decrease with an increase in testing temperature due to a change in the asphalt mix’s viscoelastic properties. A similar trend is reported by Ding et al. [61] and Huang et al. [62]. Moreover, the values for Series A are the highest among all the mixes for all testing temperatures, followed by Series B. It indicates that the minimum content of fibre provides maximum stiffness values. Also, the stiffness values for Series A and B are greater than those of the conventional HMA mix and the other modified mixes. Moreover, there is a drastic drop in the stiffness from 12,000 MPa to around half when the temperature is raised from 5 °C to 15 °C.

### 3.5. Fatigue Life

#### 3.5.1. Cyclic Tensile Stress

**Figure 15:**
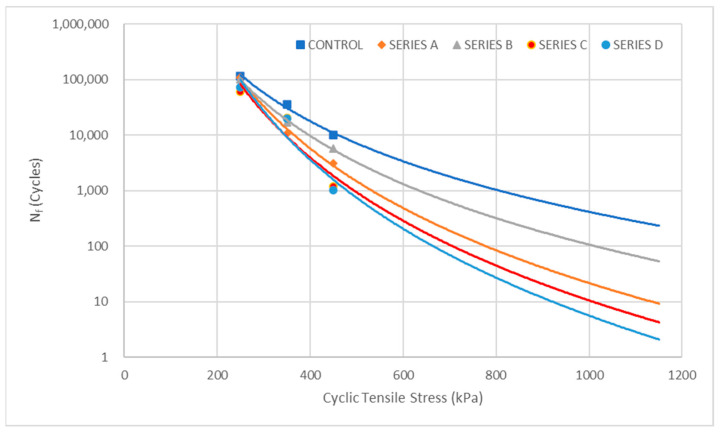
Relationship between cyclic stress levels and fatigue life.

The relationship between fatigue life (N_f_) and stress level (σ) is a fundamental aspect of asphalt pavement performance evaluation, as it provides insights into the material’s ability to resist fatigue cracking under repeated loading. The relationship between these two parameters was analyzed to understand the effect of increasing tensile stress on the performance of WTMF induced asphalt mixtures. Since fatigue models are developed with fatigue cycles as the dependent variable, the fatigue life data were plotted with stress levels on the *x*-axis and fatigue life (cycles) on the *y*-axis, as shown in Figure 15. Three samples from each mix were tested under low, medium, and high stress levels, i.e., 250, 350, and 450 kPa. The resulting trend demonstrates the inverse relationship between applied stress and fatigue resistance, where higher stress levels lead to a reduction in fatigue life. Moreover, to further evaluate this behaviour, the developed fatigue model was used to extrapolate the trend beyond the tested stress levels, predicting fatigue performance up to 100% of the ITS value. This helps in estimating the mix’s fatigue resistance under extreme loading conditions, providing valuable insights for pavement design and material optimization.

The relationship shows that as the stress level increases, the fatigue life decreases (i.e., higher stress leads to earlier failure), as reported by Peide et al. [56], Xue et al. [63], and Yuan et al. [64]. The curve generally follows a downward slope, confirming the inverse relationship between stress and fatigue resistance. Moreover, it aids in the development of fatigue models, which are essential for predicting pavement deterioration and designing long-lasting road structures. The fatigue life vs. stress level plots for different asphalt mixtures exhibit a clear trend. At low stress levels (250 kPa), all mixes converge, indicating minimal variation in fatigue performance across the different mixtures. However, as the stress level increases, the fatigue life curves start to diverge, highlighting the varying fatigue resistance of each mix. Among all tested mixes, the Control mix exhibits the highest fatigue life, as its plot remains at the topmost position in the chart across all stress levels. Following the control mix, Series B, A, and C exhibit progressively lower fatigue resistance, while Series D consistently shows the lowest fatigue life, particularly at high stress levels. Consistent with the results, recent studies have noted that fatigue life can vary by mix type, with certain preparation methods or optimal control mixes outperforming modified counterparts under identical stress conditions [63,65,66,67,68]. The increasing divergence at higher stress levels suggests that the structural integrity and fatigue resistance of some mixes degrade more rapidly under severe loading conditions. This divergence is particularly important for pavement design considerations, as it indicates that while some mixes may perform adequately under low-stress conditions, their fatigue resistance significantly diminishes under high-stress applications. The trends observed in the fatigue life plots provide valuable information for selecting optimal mix designs to enhance pavement performance.

The fatigue life increments of asphalt mixtures are highly dependent on the applied cyclic tensile stress levels. As the stress level increases, the asphalt mix undergoes greater strain accumulation, leading to accelerated fatigue damage and a shorter service life. This relationship follows a well-established trend in fatigue behaviour, where materials subjected to higher stress amplitudes experience a faster rate of microcrack initiation and propagation, ultimately reducing their fatigue resistance. The fatigue life data for different asphalt mixtures at low (250 kPa), medium (350 kPa), and high (450 kPa) cyclic tensile stress levels are presented in Table 5. As anticipated, the results indicate a progressive decrease in fatigue life with increasing stress levels for all mixtures.

The table above shows that at low stress (250 kPa), the control mix exhibits the highest fatigue life (115,529 cycles), while Series C has the lowest value (59,473 cycles). As stress increases to medium (350 kPa), fatigue life significantly decreases across all mixes, with Series A experiencing the sharpest drop (~90.2%). At high stress levels (450 kPa), the fatigue life further declines, with Series D exhibiting the lowest fatigue resistance (1012 cycles). The control mix remains the most fatigue-resistant, showing a gradual reduction in fatigue life compared to modified mixes. Series C and D, in contrast, display the highest fatigue reduction (~94%) from medium to high stress, indicating their susceptibility to fatigue cracking under extreme loading conditions. These findings emphasize the importance of stress-dependent fatigue behaviour in selecting asphalt mixtures for pavement applications. The observed divergence in fatigue performance at high stress levels highlights the need for optimized mix designs to enhance fatigue resistance under severe loading conditions.

Empirical models based on cyclic tensile stress are developed for the prediction of fatigue life. The most commonly accepted stress-based fatigue model for a given asphalt mixture can be characterized by Equation (6), where $N_{f}$ is the number of cycles to failure, σ is the applied loading level, and K and m are the material-dependent constants related to the material properties. These models usually follow a power–law relationship. [56,65,69,70]. The obtained cyclic tensile stress-based fatigue prediction models for the current study are presented in Table 6. The coefficient of determination (R^2^), which describes the relationship between applied cyclic tensile stress and fatigue life of each mixture, is also determined. A higher value of R^2^ indicates a significant correlation between applied stress and the resulting fatigue life of the specific mix.

(6) $$N_{f}=K{\left(\sigma \right)}^{-m}$$

Parameter *m* can express the attenuation rate of the asphalt mixture’s fatigue life when test stresses range from lower levels to higher levels. A bigger absolute value of parameter *m* means faster attenuation of fatigue life, and the fatigue life is more sensitive to the applied stress levels. It can be seen in Table 5 above that the differences in parameter *m* corresponding to various asphalt mixtures were not large. On the other hand, A higher *K* value indicates a material with higher fatigue resistance, meaning it can endure more loading cycles before failure at a given stress level. Higher *K* values indicate better fatigue performance.

#### 3.5.2. Resilient Micro-Strain

Resilient micro-strain (Rµε) is a critical parameter in fatigue analysis, as it represents the recoverable strain experienced by the asphalt mixture under cyclic loading. Unlike permanent strain, which accumulates over time and leads to material failure, resilient micro-strain reflects the elastic response of the mix, providing insights into its flexibility and fatigue resistance. In ITFT, resilient micro-strain is measured at different stress levels to establish a strain-based fatigue relationship, which is commonly used in empirical fatigue models. A higher resilient micro-strain typically indicates a more flexible mix, which may perform better under repeated loading but could also be more prone to fatigue cracking depending on the material composition.

Rµε was assessed during the ITFT by monitoring the recoverable horizontal deformation of the specimens under cyclic loading. During each loading cycle, the deformation response was recorded using the test system’s displacement measurement setup. The resilient micro-strain was calculated by dividing the recoverable horizontal deformation by the specimen diameter and expressing the value in micro-strain units (µε). For each mix and stress level, the average resilient micro-strain was determined from replicate specimens and used for subsequent fatigue analysis.

**Figure 16:**
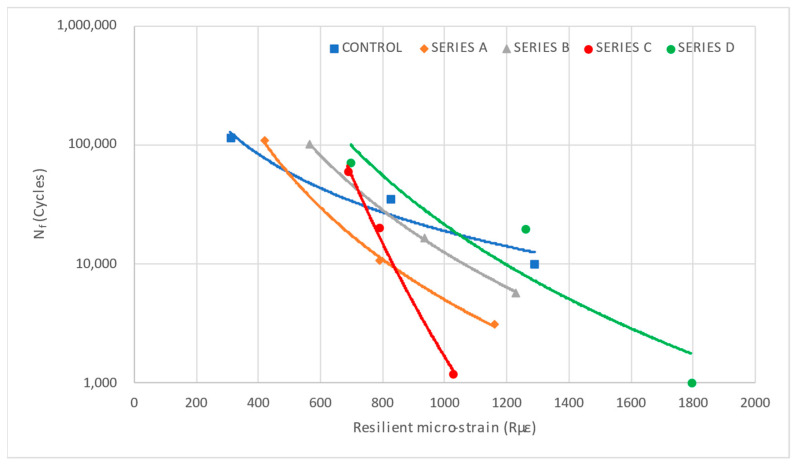
Relationship between resilient micro-strain and fatigue life.

The relationship between Rµε and fatigue life is essential for predicting pavement performance. Asphalt mixtures with lower resilient micro-strain tend to exhibit higher fatigue life, as they experience less elastic deformation under loading, reducing the likelihood of crack initiation. This study investigates the variation in resilient micro-strain across different mix types and stress levels and its correlation with fatigue cycles to develop a comprehensive fatigue prediction model for each mix. This relationship is shown in Figure 16. It presents a semi-log plot of fatigue life against resilient micro-strain. The trend confirms an inverse power–law relationship, where an increase in micro-strain results in a reduction in fatigue life. This aligns with the general fatigue behaviour of asphalt mixtures, where higher strain levels accelerate crack initiation and propagation, ultimately leading to failure in fewer cycles [71,72,73].

Table 7 presents the Rµε values for each mix type at varying stress levels. As seen in the table, the control mix exhibits the lowest resilient micro-strain values across all stress levels, indicating its superior ability to resist deformation under cyclic loading. In contrast, Series D consistently shows the highest resilient micro-strain values, suggesting increased flexibility but also greater susceptibility to fatigue failure. The micro-strain values generally increase with higher stress levels for all mix types, confirming that greater applied stress leads to higher elastic strain responses. A comparison across mix types highlights significant differences in strain behaviour. The control mix demonstrates lower strain magnitudes, aligning with its superior fatigue resistance. Series A and Series B exhibit moderate strain values, while Series C and Series D show higher strain responses, implying reduced structural integrity under repeated loading conditions. These trends are consistent with recent literature demonstrating that fibre effects on fatigue are strongly dosage- and dispersion-dependent; when fibre type, content, or mixing are not optimal, modified mixes can show reduced fatigue life relative to control mixes [10,71,72,73,74].

The basic strain-based fatigue model is represented in Equation (7), where $N_{f}$ is the number of cycles to failure, ε is the resilient micro-strain, and K and n are the material-dependent constants related to the material properties [70,75,76]. These models usually follow a power–law relationship. Alongside developing cyclic tensile stress-based fatigue life prediction models, another set of models based on Rµε was also developed. The obtained Rµε-based fatigue prediction models are presented in Table 8. The coefficient of determination (R^2^), which describes the relationship between Rµε and fatigue life of each mixture, is also determined. A higher value of R^2^ indicates a significant correlation between Rµε and the resulting fatigue life of the specific mix.

(7) $$N_{f}=K{\left(\varepsilon \right)}^{-n}$$

Parameter *n* can express the attenuation rate of asphalt mixture fatigue life when Rμε ranged from lower levels to higher levels. A bigger absolute value of parameter *n* means faster attenuation of fatigue life, and the fatigue life is more sensitive to the Rµε. On the other hand, A higher *K* value indicates a material with higher fatigue resistance, meaning it can endure more loading cycles before failure at a given strain level. Higher *K* values indicate better fatigue performance.

The findings confirm that asphalt mixtures with lower resilient micro-strain tend to exhibit longer fatigue life, as they experience lower recoverable deformation under loading. This highlights the importance of material stiffness and elasticity in improving fatigue resistance. The control mix, with the lowest resilient micro-strain values, demonstrates superior fatigue performance, while mixes with higher strain values (Series C and D) are more prone to fatigue failure.

These results reinforce the significance of resilient micro-strain as a key parameter in fatigue modelling and pavement design. Moreover, a comparison of R^2^ values indicates that both stress- and strain-based fatigue models exhibit high predictive capability, with no consistent dominance of either approach across all mix types. These developed predictive models can contribute to estimating pavement performance under various stress conditions. Overall, no single approach consistently outperformed the other across all mixtures. This indicates that both stress- and strain-based formulations are suitable for fatigue life prediction of WTMF-modified asphalt mixtures, and their combined use provides a more comprehensive understanding of fatigue behaviour.

#### 3.5.3. Permanent Deformation

**Figure 17:**
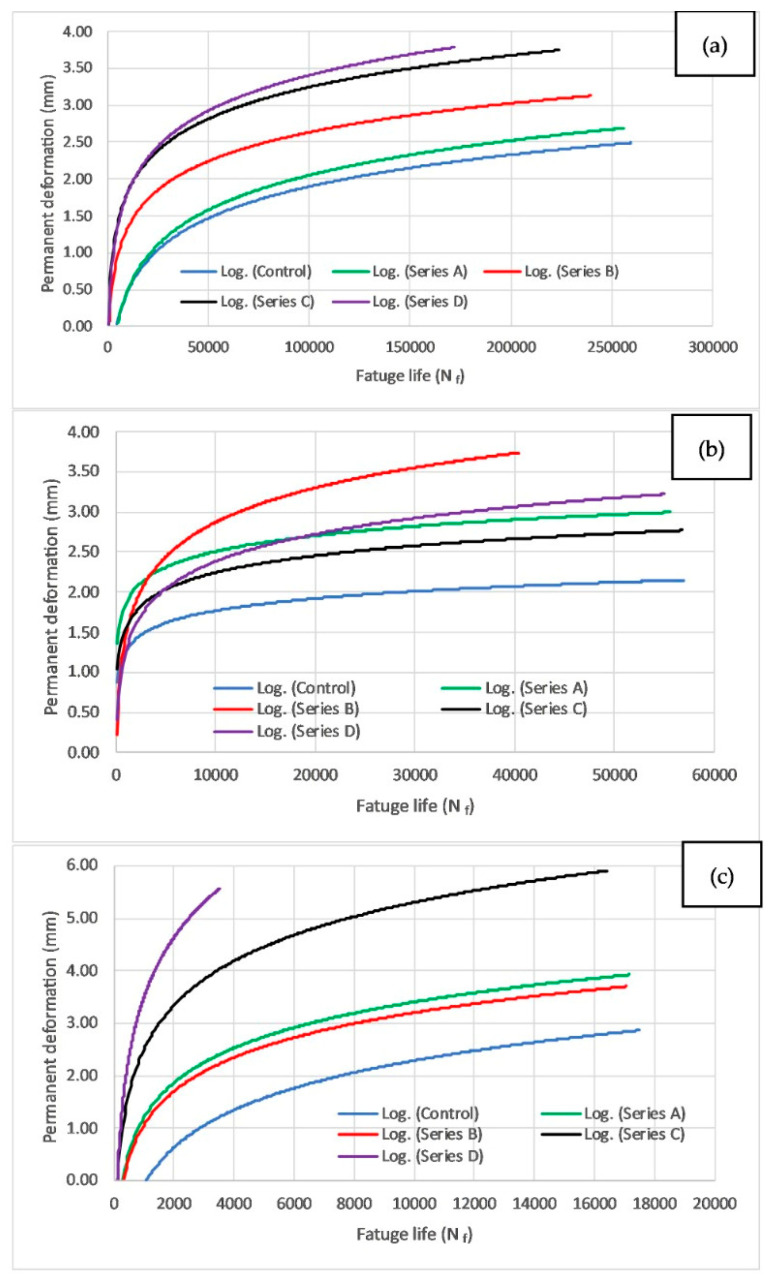
Fatigue life vs. permanent deformation at (**a**) low, (**b**) medium, (**c**) high stress levels.

**Figure 18:**
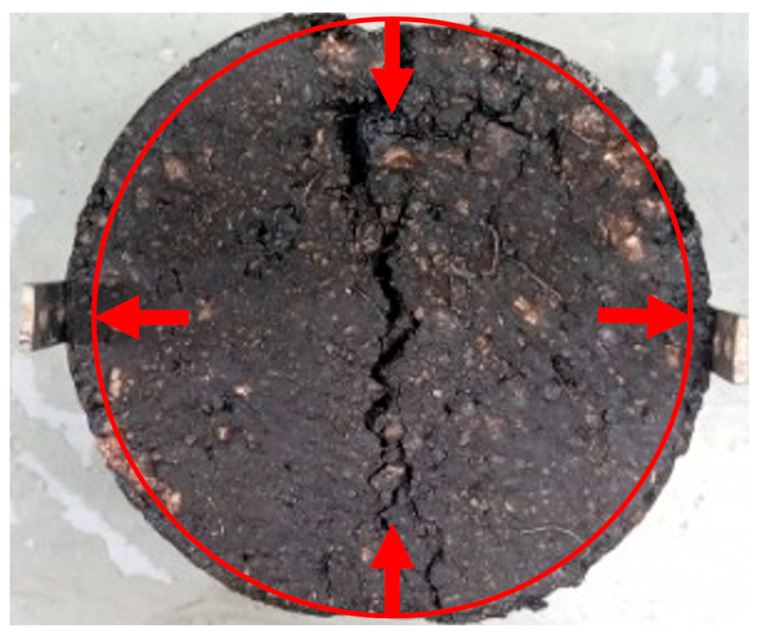
Deformed sample after ITFT with deformation strips attached. Arrows show the direction of load application during testing.

Permanent deformation, commonly referred to as rutting, is a critical distress mechanism in asphalt pavements, particularly under repeated cyclic loading. In this study, the accumulation of permanent deformation was evaluated across different mix types at three stress levels (250 kPa, 350 kPa, and 450 kPa). The relationship between fatigue life (N_f_) and permanent deformation was analyzed using three separate plots, i.e., Figure 17a–c, where the *x*-axis represents the number of cycles to fatigue failure (log scale) and the *y*-axis represents the permanent deformation (mm). The fatigue life values cited in the text refer to the minimum and maximum experimental values within each stress level used to define the logarithmic trend shown in these figures. These plots provide a framework for predicting permanent deformation based on fatigue life. It can be observed that the initial (short-term) phase is very short, where rapid deformation takes place with compaction in the specimens due to load repetition. The second (long-term) phase shows a relatively constant rate of deformation with the application of load cycles. Since WTMF acted as a binding agent due to its high aspect ratio, no unstable behaviour or sudden rupture was observed in the asphalt specimens, even when the samples failed at high fatigue cycles. Hence, no abrupt change in the deformation at the end of the test could be observed in these plots. The same phenomenon can be observed in Figure 18, which shows a deformed sample after ITFT that is still unsplit, and the two halves are in contact due to the reinforcing effect of WTMF usage. The red line shows the original alignment, and arrows represent the inward and outward direction of load application.

Table 9 presents the mean and standard deviation values for fatigue life (N_f_) and permanent deformation recorded at low, medium, and high stress levels for each mix type. The high scatter observed in fatigue life results is characteristic of asphalt fatigue testing and reflects the inherent variability of crack initiation and propagation under cyclic loading. N_f_ for all mixes decreased substantially as the stress level increased, with the most pronounced drop seen in Series C and Series D at high stress (1186 ± 1084 cycles and 1012 ± 1299 cycles, respectively). At low stress, the control mix achieved the highest mean N_f_ (115,529 ± 116,352 cycles), but the large standard deviation indicates high variability among replicates. Series A and B also exhibited high variability, particularly at medium stress, while Series C and D showed relatively lower mean fatigue lives overall. Permanent deformation generally increased with stress level for most mixes, though Series A and Series C exhibited slightly reduced deformation at high stress compared to medium stress. Series B and Series D consistently recorded higher deformation values, suggesting reduced resistance to rutting compared to the control.

At the low stress level, fatigue life values range between 15,487 and 249,479 cycles, while permanent deformation varies from 1.14 mm to 3.62 mm. The control mix consistently exhibits the highest fatigue life with the least deformation, reinforcing its superior resistance to both fatigue cracking and rutting. Conversely, Series C and D demonstrate the lowest fatigue life and highest permanent deformation values, indicating weaker performance under cyclic loading. Among the modified mixes, Series A and B perform moderately well, but Series A shows slightly lower deformation than Series B at similar fatigue life levels. The plotted curves for this stress level remain relatively close to one another, indicating that at lower stress levels, the variation in permanent deformation across the mixes is not highly pronounced (see Figure 17a).

At the medium stress level (Figure 17b), the fatigue life reduces significantly, ranging from 690 to 56,915 cycles, while permanent deformation varies between 1.64 mm and 3.56 mm. The control mix continues to show superior performance, demonstrating higher fatigue life and lower permanent deformation compared to the other mix series. However, an interesting trend is observed in this stress level: Series C outperforms Series D in terms of deformation resistance, despite its lower fatigue life. Series B and Series A show moderate performance, but Series B has slightly higher permanent deformation than Series A at similar fatigue life values. The increasing divergence in the plotted curves suggests that as the stress level increases, mix designs respond differently to fatigue loading, leading to larger variations in permanent deformation.

At the high stress level (Figure 17c), fatigue life further decreases, with values ranging from 237 to 17,510 cycles, while permanent deformation increases significantly, ranging between 1.04 mm and 5.00 mm. The control mix remains the best-performing mixture, exhibiting the longest fatigue life with the least permanent deformation. Notably, at this high stress level, the divergence between the plotted curves is the most pronounced, indicating that different mixtures respond very differently to extreme cyclic loading. Series D exhibits the highest permanent deformation (up to 5.01 mm), while Series C follows closely behind, confirming their susceptibility to rutting at higher stress levels. Series B and A show intermediate behaviour, with Series B slightly outperforming Series A in terms of rutting resistance.

Looking at the data and plots given above, an overall analysis showed that the control mix consistently demonstrates the highest fatigue life and lowest permanent deformation across all stress levels, indicating strong resistance to both fatigue and rutting. Although studies directly confirming that control mixes outperform the modified ones in both fatigue life and permanent deformation across all stress levels are limited, they have shown that steeper stiffness does not always translate to superior fatigue resistance [77] and that enhancements in rutting resistance may not always coincide with improved fatigue life [78]. These findings align with the current study’s observation that the control mix demonstrated both the highest fatigue life and the lowest permanent deformation. At low stress levels, the difference in permanent deformation among mix types is minimal, suggesting similar performance under lower loads. As stress levels increase, the permanent deformation of modified mixes varies significantly, leading to larger divergences in fatigue life vs. deformation curves. At high stress levels, some modified mixes (especially Series C and D) exhibit significantly lower fatigue life and greater permanent deformation, highlighting their poor rutting resistance under heavy loading conditions. Series A and B show balanced performance, but Series B tends to experience greater deformation than Series A at comparable fatigue lives.

The relationship between fatigue life and permanent deformation is critical for asphalt mix design and pavement performance evaluation. A mix with high fatigue life but excessive permanent deformation may still fail prematurely due to rutting, while a mix with low fatigue life but minimal deformation may not be practical for long-term use. Therefore, an optimal balance between fatigue resistance and rutting performance is essential when selecting asphalt mixtures for different traffic and loading conditions. These findings suggest that for high-traffic applications, the control mix remains the most suitable choice, whereas Series A and B may serve as alternative options depending on specific performance criteria. However, Series C and D demonstrate limited applicability under high-stress conditions, requiring further modifications or adjustments in mix design to enhance their rutting and fatigue resistance.

**Figure 19:**
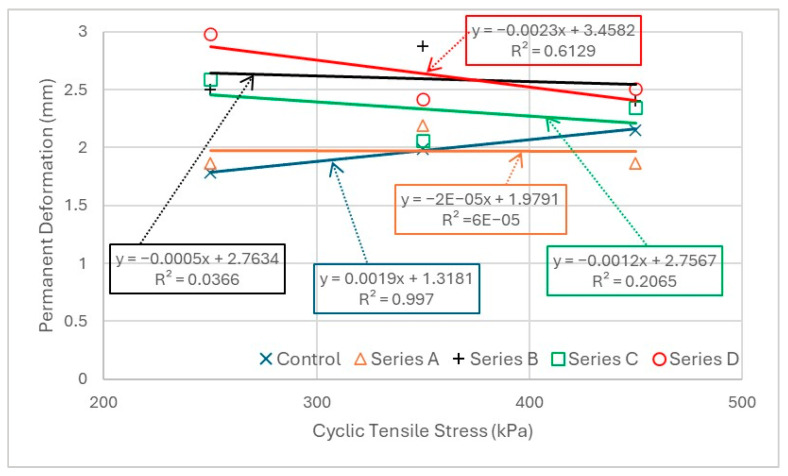
Relationship between maximum permanent deformation and stress level.

The relationship between cyclic tensile stress levels and permanent deformation is critical in assessing the rutting susceptibility of asphalt mixtures under repeated loading. To evaluate this behaviour, permanent deformation values are plotted against different stress levels for each mix type, as depicted in Figure 19. The results indicate that permanent deformation values at the low stress level (250 kPa) are more widely spread, ranging from 3.0 mm to 1.7 mm across different mixes. In contrast, at the high stress level (450 kPa), the spread is narrower, ranging from 2.6 mm to 1.8 mm. This suggests that at lower stress levels, the permanent deformation behaviour varies more significantly between mixes, whereas at higher stress levels, the deformation response becomes more uniform across all mixes. Consistent with recent observations, lower stress levels produced more gradual and varied permanent deformation responses among mixes, whereas at higher stress levels, deformation curves tended to converge—suggesting more uniform behaviour across mix types [79,80].

The statistical correlation, represented by R^2^ values, further highlights the relationship between stress level and permanent deformation. For most mix series, the R^2^ values are very low, indicating a weak correlation. However, the control mix exhibits an exceptionally high R^2^ value of 0.997, suggesting that its deformation behaviour follows a well-defined trend with stress level. The low R^2^ values for the modified mixes indicate greater variability in their response to stress, which could be attributed to differences in material composition, structural integrity, or interaction effects between fatigue damage and rutting.

From an engineering perspective, the higher spread of deformation at low stress levels may indicate greater sensitivity to mix design variations, requiring careful optimization of asphalt mixtures. The narrowing of deformation spread at higher stress levels suggests that permanent deformation becomes more predictable as stress increases, likely due to the dominant effect of accumulated damage. The low R^2^ values, except for the control mix, suggest that permanent deformation may be influenced by additional factors beyond just applied stress, such as binder properties, aggregate interlock, and internal structural changes under cyclic loading. These findings highlight the importance of considering both stress-dependent deformation trends and statistical reliability when assessing the long-term rutting resistance of asphalt mixtures.

## 4. Conclusions

This study utilized waste tyre metal fibre (WTMF) in hot mix asphalt (HMA) to evaluate the Marshall and volumetric properties, moisture damage susceptibility, the indirect tensile stiffness modulus and the indirect tensile fatigue failure. The fatigue life assessment was done by cyclic tensile stress, resilient micro-strain, and permanent deformation analyses. Moreover, statistical models were developed for predicting the fatigue life of WTMF-modified HMA mixtures that were not directly tested in the laboratory. The following are the conclusions drawn in accordance with the findings of the experimental investigation.

- WTMF characterization revealed that the fibres possess an irregular, rough surface texture with microcracks and pits, coupled with a high aspect ratio that promotes effective stress transfer and crack-bridging. Spectroscopic analysis confirmed a predominant presence of carbon and iron, indicating that the combination of this rough microstructure and chemical composition can enhance mechanical interlocking and overall performance when incorporated into asphalt mixtures in controlled dosages.
- Marshall and volumetric properties indicated that the optimum binder content for WTMF-modified HMA was, on average, 5% higher than that of the conventional mix, attributable to increased surface area and air voids. However, the addition of WTMF reduced Marshall stability, increased flow, and lowered Marshall quotient, likely due to fibre conglomeration during mixing, which also adversely affected porosity and bulk specific gravity, showing the importance of the dosage-dependent nature of WTMF.
- ITS and TSR results showed that wet samples generally exhibited lower ITS values than dry samples, with the control mix recording the highest ITS. However, WTMF-modified mixes demonstrated superior moisture resistance, as indicated by TSR values exceeding 80% in almost all cases. The highest TSR was achieved with 0.75% WTMF, showing an 11% increase over the control mix, while only the 1.50% WTMF mix experienced a slight 2.4% reduction. Although the control had the maximum ITS, the WTMF mixes exhibited better strength retention after moisture exposure.
- Stiffness modulus results indicated that the mix containing 0.375% WTMF achieved the highest modulus values across all testing temperatures, with the peak recorded at 5 °C due to the viscoelastic nature of bitumen. Compared to the control mix, the modified mix showed up to 70% higher stiffness at low temperatures, with the relative difference increasing to more than double at higher temperatures. For all mixes, stiffness decreased with increased temperature, with an 84% reduction observed in the control and 80% in the 0.375% WTMF mix, confirming the reinforcing effect of fibres in improving mixture stiffness.
- The fatigue life analysis indicated that while the control mix achieved the highest life at low stress (115,529 cycles), it suffered steep reductions of 69.6% from low to medium stress and 71.1% from medium to high stress. The evaluation, conducted using cyclic tensile stress, resilient micro-strain, and permanent deformation parameters, demonstrated that moderate incorporation of WTMF enhanced strain tolerance, but excessive fibre content increased permanent deformation under high stress, potentially compromising the long-term performance of HMA.

This study has demonstrated the potential of incorporating waste tyre metal fibres (WTMFs) into asphalt mixtures to improve the performance of HMA mixes, thereby promoting sustainable pavement engineering practices. However, it is noteworthy that the benefits of WTMF usage depend on its dosage and targeted performance (e.g., moisture resistance, stiffness), and that the optimum range of WTMF found in this study is in the range of 0.375 to 0.75%. The results highlight the significance of adopting innovative, waste-derived materials in road construction to address both performance and environmental challenges. Moreover, by integrating waste management with pavement engineering, this study paves the way for innovative, eco-friendly solutions that align with global sustainability goals and offer long-term value for infrastructure development. Based on the findings, future studies should investigate a broader range of fibre contents, aspect ratios, and geometries to determine the most effective configurations for improving the asphalt’s mechanical performance indicators. Also, long-term field trials are recommended to assess the in-service behaviour of WTMF-modified asphalt mixtures under diverse climatic conditions and traffic loadings, with a focus on ageing, fatigue resistance, moisture susceptibility, and rutting performance over extended service life.

In general, the addition of waste tyre metal fibres (WTMFs) shows a distinct dosage effect on the performance of the asphalt mixture. In low dosages, WTMF enhances the waterproofing properties, as well as the stiffness of the mixture, without compromising the volumetric properties. However, higher dosages of WTMF reduce the stability, fatigue, and strength of the mixture, mainly because of the reduction in cohesion and the lack of workability of the mixture. This demonstrates the importance of WTMF optimization as a means of increasing strength while preventing the lowering of mechanical properties.

## Figures and Tables

**Table 1:** Overview of existing literature on fibre-reinforced asphalt mixtures illustrating the range of fibre contents and performance tests considered in previous studies.

Reference	Modifier Type	Material	Dosage Range	Basis of Content
Messaoud et al. (2022) [18]	Metal/Carbon-based	Steel fibre/Graphite powder	0.2–1.2%; 1–30%	Mix/Volume
Shafi Ullah et al. (2022) [6]	Carbon fibre	Carbon fibre	0.25%	Mix
Wang et al. (2022) [19]	Metal/Carbon-based	Nickel/Carbon fibre/Graphene	0.1–19.1%	Mix
Hanwen et al. (2022) [20]	Metal/Carbon-based	Steel wool/Graphite	2–8%; 10–20%	Volume of asphalt
Jia-Liang Le et al. (2022) [21]	Nanomaterial	GNP/Taconite	6%	Binder weight
Zhenxia Li et al. (2022) [22]	Carbon-based	Carbon fibre/Graphite	0.3–0.5%; 20–40%	Mix
Shaffie et al. (2023) [23]	Metal fibre	Steel fibre	0–0.7%	Total mix
Ying-Yuan et al. (2022) [24]	Carbon-based fibre	Carbon fibre/Graphene	0.1–0.3%; 0.5–2.0%	Mix/Binder
Zejiao Dong et al. (2022) [25]	Carbon fibre	Carbon fibre	0.35%	Mix
Gürer et al. (2022a) [5]	Polymer/Carbon-based	SBS/Carbon fibre/Carbon black	0.3–14%	Binder
Gürer et al. (2022b) [26]	Metal/Carbon-based	Metal fibre/Carbon fibre	~2.16%	Binder
Luana et al. (2023) [27]	Nanomaterial/Metal fibre	CNT/Steel wool	1%; ~1.0%	Binder weight

**Table 2:** Properties of aggregates and bitumen used in the research.

Property	Aggregates	JKR Specification	Bitumen	JKR Specification
Penetration	-	-	63 dmm	60/70 or 80/100
Ductility	-	-	135 cm	>50 cm
Softening point	-	-	49.6 °C	35–70 °C
Specific gravity	2.60	2.50–3.00	1.03	0.97–1.02
Water absorption	4%	≯2%	-	-
Los Angeles abrasion	21.5%	≯25%	-	-
Flakiness	21.4%	≯25%	-	-
Elongation	22.7%	≯25%	-	-
Crushing value	24%	≯25%	-	-

**Table 3:** EDS quantitative analysis.

Element	Atomic Percent	Weight Percent
Carbon	57.32	28.99
Copper	2.70	7.23
Iron	21.97	51.65
Oxygen	18.01	12.13
Total	100	100

**Table 4:** Marshall parameters and volumetrics with different percentages of WTMF and OBC.

Series	OBC (%)	G*_mb_*	Porosity (%)	VMA (%)	VFB (%)	Stability (kN)	Flow (mm)
Control	4.6	2.3316	4.03	14.45	72.23	15.38	2.21
Series A	4.9	2.3154	4.30	15.31	71.98	12.89	2.87
Series B	5.0	2.3111	4.47	15.47	71.14	12.69	3.84
Series C	4.9	2.3033	4.79	15.75	69.60	12.33	3.94
Series D	4.5	2.3091	5.10	15.18	66.48	12.10	4.10

**Table 5:** Average fatigue life increments and their subsequent reduction based on stress levels.

Mix Type	Cyclic Tensile Stress Level			Percent Fatigue Life Reduction	
	Low (250 kPa)	Medium (350 kPa)	High (450 kPa)	Low to Medium Stress	Medium to High Stress
Control	115,529	35,120	10,153	69.6	71.1
Series A	108,990	10,693	3117	90.2	71.9
Series B	102,473	16,736	5761	83.7	65.6
Series C	59,473	20,184	1186	66.0	94.1
Series D	71,437	19,661	1012	72.5	94.9

**Table 6:** Fatigue life prediction models based on cyclic tensile stress.

**Mix Type**	Model	Parameter K	Parameter m	R^2^
Control	$N_{f}=8\times 10^{14}\sigma^{-4.104}$	8 × 10^14^	−4.104	0.996
Series A	$N_{f}=4\times 10^{19}\sigma^{-6.094}$	4 × 10^19^	−6.094	0.999
Series B	$N_{f}=6\times 10^{16}\sigma^{-4.924}$	6 × 10^16^	−4.924	0.999
Series C	$N_{f}=3\times 10^{20}\sigma^{-6.472}$	3 × 10^20^	−6.472	0.943
Series D	$N_{f}=2\times 10^{20}\sigma^{-6.474}$	2 × 10^20^	−6.474	0.918

**Table 7:** Resilient micro-strain measurements based on stress levels.

Mix Type	Cyclic Tensile Stress Level		
	Low (250 kPa)	Medium (350 kPa)	High (450 kPa)
Control	308.89	825.00	1288.67
Series A	417.00	788.67	1160.67
Series B	562.00	932.00	1226.00
Series C	686.67	790.33	1028.00
Series D	696.50	1258.33	1793.50

**Table 8:** Fatigue life prediction models based on Rµε.

**Mix Type**	Model	Parameter K	Parameter n	R^2^
Control	$N_{f}=1\times 10^{9}\varepsilon^{-1.622}$	1 × 10^9^	−1.622	0.985
Series A	$N_{f}=1\times 10^{14}\varepsilon^{-3.489}$	1 × 10^14^	−3.489	0.999
Series B	$N_{f}=1\times 10^{15}\varepsilon^{-3.677}$	1 × 10^15^	−3.677	0.999
Series C	$N_{f}=6\times 10^{32}\varepsilon^{-9.841}$	6 × 10^32^	−9.841	0.991
Series D	$N_{f}=9\times 10^{15}\varepsilon^{-3.895}$	9 × 10^15^	−3.895	0.910

**Table 9:** Mean and standard deviation values of fatigue life and deformation at predefined stress levels for each mix type.

Mix Series	Low Stress Level		Medium Stress Level		High Stress Level	
	N_f_ (Cycles)	Deformation (mm)	N_f_ (Cycles)	Deformation (mm)	N_f_ (Cycles)	Deformation (mm)
Control	115,529 ± 116,352	1.78 ± 0.64	35,116 ± 24,636	1.98 ± 0.23	10,153 ± 6620	2.15 ± 0.75
Series A	108,990 ± 101,884	1.91 ± 0.62	10,693 ± 16,322	2.19 ± 0.56	3117 ± 3518	1.86 ± 1.09
Series B	102,473 ± 80,756	2.50 ± 0.56	16,736 ± 19,724	2.88 ± 0.96	5761 ± 5479	2.41 ± 1.08
Series C	59,473 ± 73,057	2.59 ± 0.82	20,184 ± 31,652	2.06 ± 0.64	1186 ± 1084	2.35 ± 1.15
Series D	71,437 ± 62,646	2.98 ± 0.66	19,661 ± 22,409	2.42 ± 0.72	1012 ± 1299	2.51 ± 2.17

## Data Availability

Data will be made available upon reasonable request.
